# False Positive Radioiodinated Metaiodobenzylguanidine (^123^I-MIBG) Uptake in Undifferentiated Adrenal Malignant Tumor

**DOI:** 10.1155/2015/164280

**Published:** 2015-03-30

**Authors:** Hee Soo Jung, Seok Jun Moon, Yun Mi Kim, Hye Rim Kang, Seok Mo Lee, Soo Jin Jung, Seok Jin Choi, Tae Kyoon Kim, Min Jeong Kwon, Jeong Hyun Park, Soon Hee Lee

**Affiliations:** ^1^Department of Internal Medicine, College of Medicine, Inje University, Busan 614-735, Republic of Korea; ^2^Department of Nuclear Medicine, Busan Paik Hospital, College of Medicine, Inje University, Busan 614-735, Republic of Korea; ^3^Department of Pathology, Busan Paik Hospital, College of Medicine, Inje University, Busan 614-735, Republic of Korea; ^4^Department of Radiology, Busan Paik Hospital, College of Medicine, Inje University, Busan 614-735, Republic of Korea; ^5^Division of Endocrinology and Metabolism, Department of Internal Medicine, College of Medicine, Inje University, Busan 614-735, Republic of Korea

## Abstract

^123^I-Metaiodobenzylguanidine (^123^I-MIBG) scintigraphy is a widely used functional imaging tool with a high degree of sensitivity and specificity in diagnosis of pheochromocytoma. However, rare cases of false positive reactions have been reported. A 67-year-old male patient was admitted with epigastric pain. Abdominal computed tomography (CT) revealed a heterogeneous left adrenal mass 6 cm in diameter; following hormone testing, ^123^I-MIBG scintigraphy was performed to determine the presence of pheochromocytoma, which confirmed eccentric uptake by a large left adrenal gland mass. Chest CT and PET-CT confirmed metastatic lymphadenopathy; therefore, endobronchial ultrasound transbronchial needle aspiration was performed. Metastatic carcinoma of unknown origin was suspected from a lymph node biopsy, and surgical resection was performed for definitive diagnosis and correction of excess hormonal secretion. A final diagnosis of undifferentiated adrenal malignant tumor was rendered, instead of histologically malignant pheochromocytoma, despite the uptake of ^123^I-MIBG demonstrated by scintigraphy.

## 1. Introduction

Metaiodobenzylguanidine (^123^I-MIBG) is an analog of norepinephrine that accumulates in neurosecretory vesicles via the norepinephrine transporter and passive diffusion and is widely used in diagnosis and localization of pheochromocytoma [1].

A recent meta-analysis showed that the sensitivity of ^123^I-MIBG scintigraphy is 94% (95% CI, 91–97%) with a specificity of 92% (95% CI, 87–98%) for pheochromocytoma [2]. Rare cases of false positive uptake have been reported in hepatocellular carcinoma, adrenocortical carcinoma, malignant lymphoma, and adrenocortical adenoma [3–6]. Pheochromocytoma results in hypertension in 0.5% of patients and adrenal incidentaloma in 4% of cases; approximately 24% of neuroendocrine tumors are related to gene mutations associated with a hereditary syndrome [7, 8]. The patient in the present case report was histologically diagnosed following surgical resection as having undifferentiated adrenal malignant tumor that showed positive uptake on ^123^I-MIBG scintigraphy, which is intended to detect pheochromocytoma.

## 2. Case Presentation

A 67-year-old man was admitted with epigastric pain. At the time of the admission, blood pressure was 100/60 mmHg, pulse was 92 beats/min, respiratory rate was 20 breaths/min, and temperature was 37.2°C. The patient was referred to our hospital with general weakness and a weight loss of 10 kg for the last 3 months. Mild epigastric tenderness was revealed during physical examination.

His laboratory tests revealed anemia with hemoglobin of 7.6 g/dL and leukocytosis with white blood cell count of 18,380/mm^3^. A comprehensive metabolic blood panel revealed a low albumin level of 2.5 g/dL, low sodium of 130 mEq/L, increased AST/ALT of 131/117 IU/L, and increased alkaline phosphatase of 539 IU/L. Renal functions were normal.

An electrocardiogram revealed right bundle branch block, with no changes compared to the previous exam. The levels of creatine kinase MB and troponin-I were normal.

**Figure 1:**
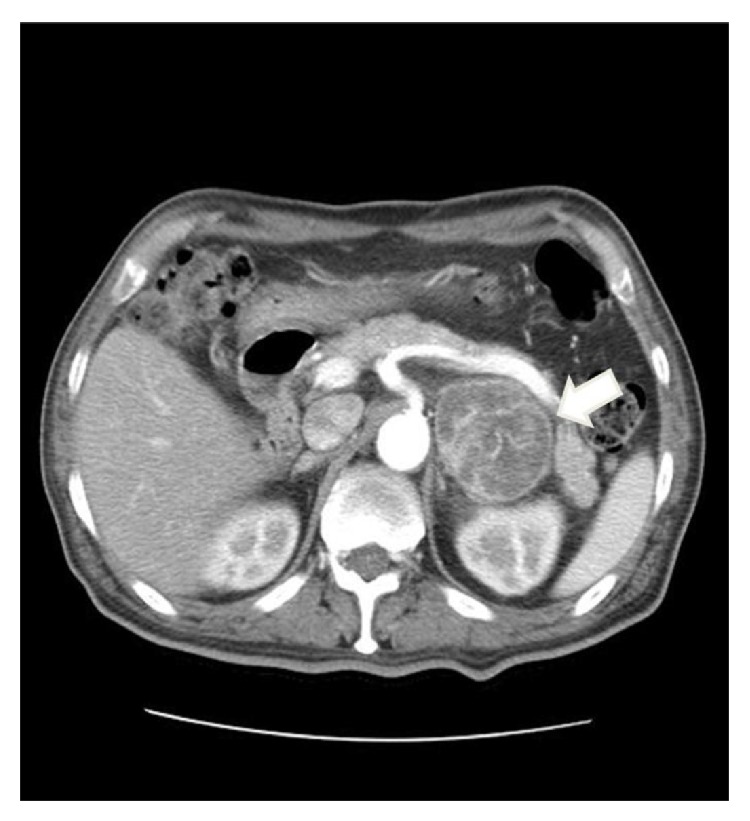
Contrast-enhanced abdominal computed tomography shows a heterogeneously enhancing left adrenal mass (4.8 × 5.7 cm) with irregular internal tumor necrosis (white arrow). There was no abnormality in the right adrenal gland (not shown).

Abdominal computed tomography (CT) revealed a heterogeneous left adrenal mass 6 cm in diameter, accompanied by hemorrhaging (Figure 1).

Hormonal testing through 24 h urine collection showed the following results: epinephrine: 4.8 *μ*g/day (range, 0~20); norepinephrine: 52.1 *μ*g/day (range, 15~80); 17-ketosteroid: 5.76 mg/day (range, 10~25); and metanephrine: 0.47 mg/day (range, 0~1.3).

**Figure 2:**
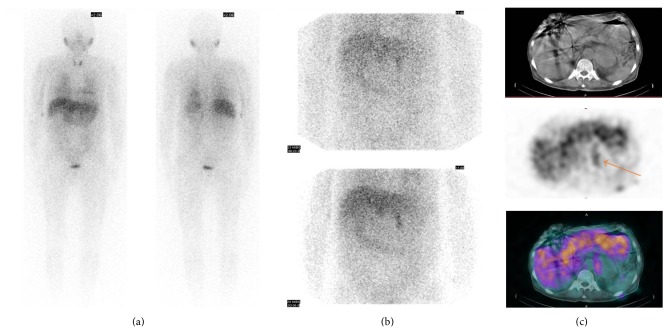
Early ^123^I-MIBG scintigraphy showed no ^123^I-MIBG uptake in suprarenal area (a). 24-hour delayed ^123^I-MIBG scintigraphy, however, showed increased ^123^I-MIBG uptake in left suprarenal area (b). On the image of ^123^I-MIBG SPECT/CT, left adrenal gland tumor showed ^123^I-MIBG uptake in the peripheral portion ((c), arrow).

The levels of urine vanillylmandelic acid (VMA), 10.42 mg/day (range, 0~8), and urine-free cortisol, 491.4 *μ*g/day (range, 55.5~286), were elevated. In addition, plasma ACTH, <1.0 pg/mL (range, 7.2~63.3), was suppressed, with normal plasma renin activity and aldosterone levels. With the suppression of ACTH and the elevation of urine-free cortisol, we performed a low-dose dexamethasone suppression test to check for Cushing's syndrome. The plasma cortisol level (6.13 *μ*g/dL) showed no suppression at the end of test. We interpreted these results as subclinical Cushing syndrome because he had no symptoms or signs of Cushing's syndrome. ^123^I-Metaiodobenzylguanidine (^123^I-MIBG) scintigraphy was performed to check for the presence of pheochromocytoma. For this purpose, 3 mCi of ^123^I-MIBG was injected and whole body images were obtained after 6 h and after 24 h. From the 24 h images, eccentric uptake by a large left adrenal gland mass was confirmed (Figures 2(a) and 2(b)). On the image of ^123^I-MIBG SPECT/CT, left adrenal gland tumor showed ^123^I-MIBG uptake in the peripheral portion (Figure 2(c)).

**Figure 3:**
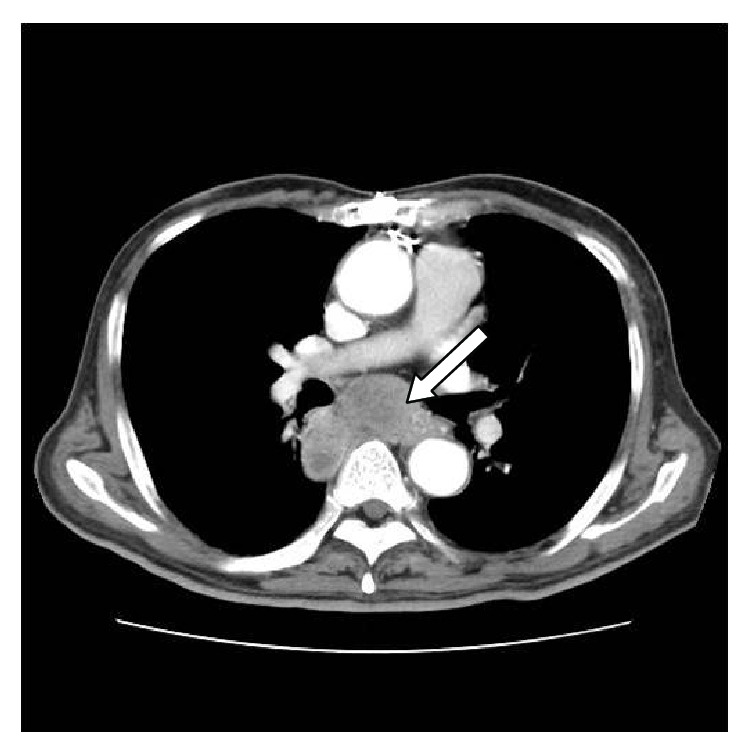
Chest computed tomography scan shows the multiple metastatic lymph nodes enlargement at subcarinal area (white arrow).

**Figure 4:**
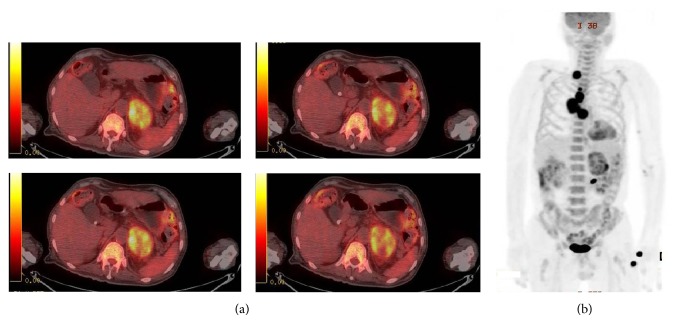
Transaxial image of ^18^F FDG PET/CT showed heterogeneously FDG-avid left adrenal gland tumor (a) and MIP of ^18^F FDG PET/CT (b) also revealed multiple FDG-avid lymphadenopathies in mediastinum and right supraclavicular area.

During preparation of surgery for suspected subclinical Cushing's syndrome and pheochromocytoma, chest X-ray and chest CT (Figure 3) confirmed metastatic lymphadenopathy of the mediastinum. PET-CT (Figure 4) and endobronchial ultrasound transbronchial needle aspiration (EBUS-TBNA) were also performed. From the lymph node biopsy obtained by EBUS-TBNA, metastatic carcinoma of unknown origin was suspected, and surgery was prepared in suspicion of malignant pheochromocytoma or adrenocortical carcinoma, accompanied by metastatic lymphadenopathy.

**Figure 5:**
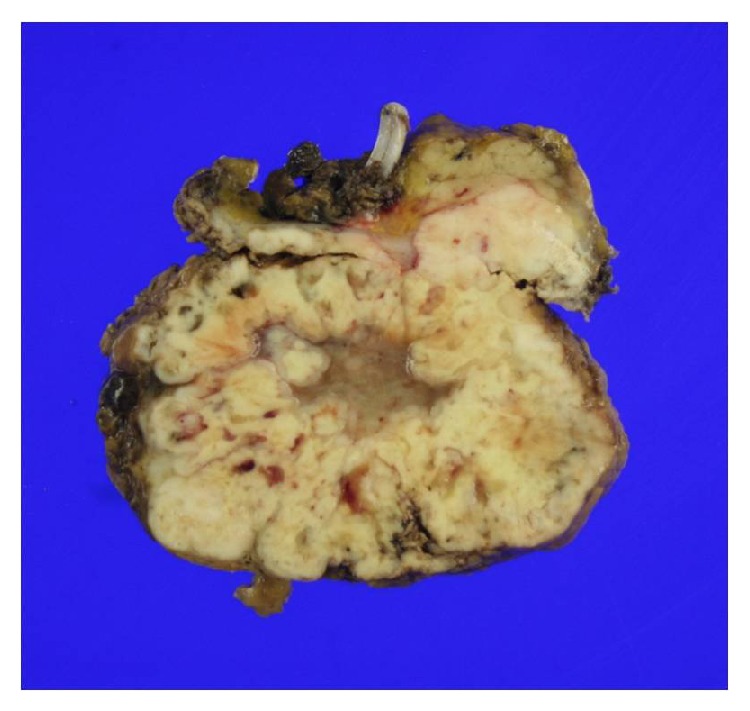
Grossly the adrenal mass is pale yellowish solid with lobular pattern and centrally myxoid degeneration and geographic necrosis.

**Figure 6:**
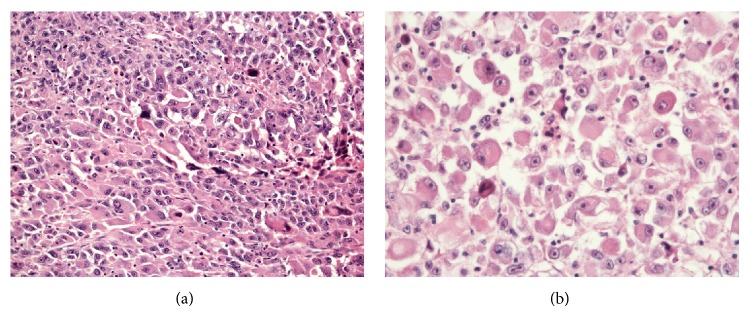
Histopathology of tumor. (a) The tumor cells are arranged in solid sheets and show pleomorphic nuclei with prominent nucleoli (H&E, ×100). (b) Some tumor cells have eccentric nuclei with abundant cytoplasm (H&E, ×200).

Starting from 2 weeks prior to the surgery, phenoxybenzamine and propranolol were administered. Laparoscopic left adrenalectomy was performed. The resected tumor measured 8.5 × 7.5 × 5.1 cm; central necrosis and hemorrhaging were present, with involvement of the resection margins (Figure 5). Microscopically, the tumor revealed a solid sheet arrangement composed of pleomorphic cells. Tumor cells had enlarged pleomorphic nuclei with prominent nucleoli and abundant cytoplasm, while some cells show rhabdoid features with abundant eosinophilic cytoplasm and eccentric nuclei (Figure 6). Pathologically, pheochromocytoma could be excluded since neuroendocrine markers (chromogranin, CD56, and NSE) were negative. The tumor was located within adrenal parenchyma with outside protruding feature in grossly. And inhibin and vimentin immunohistochemical stains were positive in tumor cells. Both antibodies are usually positive in adrenal cortical tumor. Grossly and immunohistochemically, this tumor was considered in primary adrenal tumor although it demonstrated histologically undifferentiated feature. A diagnosis of undifferentiated adrenal tumor with rhabdoid features was rendered.

Two days after tumor resection, the patient reported dyspnea and developed hypoxia; chest CT confirmed the presence of multifocal pulmonary thromboembolism, bilateral pneumonia, and pulmonary infarction. Mechanical ventilation, intensive care, and administration of antibiotics were continued, but the patient showed pneumonia aggravation, acute kidney injury, and fungemia, with no signs of improvement. The patient's legal guardian refused to continue intensive care and transferred the patient to another hospital for supportive treatment. The patient died 2 months after his initial diagnosis.

## 3. Discussion

Pheochromocytoma, along with paraganglioma, is a chromaffin neuroendocrine tumor that secretes catecholamine, which mostly manifests characteristic symptoms including headache, hypertension, and palpitation. However, approximately 10% of patients may show no symptoms, and careful evaluation is necessary, as pheochromocytoma can be life threatening if left undiagnosed [1, 7]. A diagnosis of pheochromocytoma is obtained primarily by biochemical testing, whereas if catecholamine excess is seen, imaging studies are performed in order to localize the tumor. Imaging can also be considered in cases with ambiguous results from biochemical testing and in patients with genetic disposition or previous history of pheochromocytoma [9]. Even with normal results from biochemical testing, it may be difficult to rule out pheochromocytoma, and in about 10% of pheochromocytoma and paraganglioma patients with a succinate dehydrogenase subunit B gene mutation have normal results in biochemical tests. Moreover, there have been recent reports of asymptomatic pheochromocytoma in patients who showed no excess of catecholamine during biochemical testing due to adrenal incidentaloma [10–12].

Currently, ^123^I-MIBG scintigraphy is widely used as a functional imaging tool. It has high sensitivity and specificity in diagnosis of neuroendocrine tumors, which, along with anatomical imaging tools such as CT and MRI, is recommended in the diagnosis and localization of pheochromocytoma [9]. In addition, in patients diagnosed with pheochromocytoma through biochemical testing, the combined use of MRI and ^123^I-MIBG scintigraphy has a sensitivity and positive predictive value of 100%; even in patients with symptomatic pheochromocytoma and normal urinary catecholamine metabolites, positive uptake in ^123^I-MIBG scintigraphy is seen. Taken together, this demonstrated the effectiveness of ^123^I-MIBG scintigraphy in diagnosis of pheochromocytoma [13, 14].

However, rare cases of false positive uptake in ^123^I-MIBG scintigraphy have been reported, even though the mechanism for false positive uptake remains unclear. Several explanations have been proposed in confirmed cases of false positive uptake. For example, neuroendocrine tumors with origins other than a chromaffin neuroendocrine tumor, medullary thyroid carcinoma, and carcinoid tumors can all show uptake of ^123^I-MIBG, which is believed to occur via intracellular granules through their respective amine uptake mechanisms [15]. Furthermore, in cases of lesions in the adrenal gland, in the absence of neuroendocrine tumor, false positive uptake has been reported in patients with adrenocortical adenoma and adrenal metastasis of choriocarcinoma, which may be related to the low level of ^123^I-MIBG uptake by normal adrenal gland [6, 7, 16].

The uptake of ^123^I-MIBG into cells from chromaffin neuroendocrine tumors mainly occurs via the norepinephrine transporter and passive diffusion, which is then accumulated in the secretory granules inside cells by vesicular monoamine transporters (VMATs) [1, 17]. False positive uptake of ^123^I-MIBG has been reported in large adrenocortical carcinoma, hepatocellular carcinoma, and malignant lymphoma, which is suspected to be related to increased ^123^I-MIBG uptake by passive diffusion resulting from increased blood flow [3–5]. Moreover, cases of false positive ^123^I-MIBG uptake have been reported in mast cell-infiltrated infantile hemangioma, which is believed to be caused by increased expression of VMAT2 by mast cells [18].

In this case, the false positive uptake seen on ^123^I-MIBG scintigraphy is believed to be the result of increased passive diffusion from increased blood flow and the lesion's location in the adrenal gland. Furthermore, no uptake of ^123^I-MIBG may have been seen in metastatic lymphadenopathy because, after acquiring a whole body image in the first 6 h, the subsequent topography performed after 24 h focused only on the adrenal gland.

In the present case, except for weight loss, other symptoms typical of pheochromocytoma such as headache and palpitation were absent; normal levels of catecholamine and metanephrine were found in hormonal testing, with slight elevation seen only in VMA. However, in biochemically silent cases of pheochromocytoma, instead of typical symptoms associated with catecholamine excess, only the symptoms associated with the tumor effect may be present, as seen in the present case [7]. Furthermore, since untreated pheochromocytoma can be fatal, ^123^I-MIBG scintigraphy was performed to assess the presence of pheochromocytoma.

Metastatic lymphadenopathy that was not seen in ^123^I-MIBG scintigraphy was discovered by chest CT and PET-CT, which led to suspicion of malignant pheochromocytoma. Malignant pheochromocytoma comprises 13~34% of all pheochromocytoma cases, where about one-half shows metastasis at first diagnosis, with the rest undergoing metastasis after a median time of 5.6 years [7]. Although malignant pheochromocytoma is not pathognomic, in CT it is seen as a heterogeneous mass of irregular shape, with an average size of 5 cm and accompanying necrosis [19]. In the present patient, malignancy was first suspected from CT findings and considering the size of the tumor.

The patient underwent surgical resection for definitive diagnosis and correction of excess hormonal secretion. Unfortunately, two days after surgery, the patient developed pulmonary thromboembolism, pulmonary infarction, and pneumonia, which did not improve. The patient died 2 months after his initial diagnosis. The utility of ^123^I-MIBG scintigraphy in diagnosis of pheochromocytoma is widely known and can be performed in cases where catecholamine excess is not confirmed by biochemical testing, but when clinically suspected. However, despite the fact that ^123^I-MIBG scintigraphy is an outstanding functional imaging tool, due to the remote possibility of false positive uptake, interpretation of ^123^I-MIBG scintigraphy and diagnosis of pheochromocytoma should be made with caution. When the degree of ^123^I-MIBG uptake is low-grade in relationship to a large tumor mass, then a false positive result should be entertained, particularly in the absence of typical clinical and biochemical features of pheochromocytoma.
